# Validation metrics of homogenization techniques on artificially inhomogenized monthly temperature networks in Sweden and Slovenia (1950–2005)

**DOI:** 10.1038/s41598-021-97685-7

**Published:** 2021-09-14

**Authors:** Roberto Coscarelli, Giulio Nils Caroletti, Magnus Joelsson, Erik Engström, Tommaso Caloiero

**Affiliations:** 1 National Research Council of Italy, Research Institute for Geo-Hydrological Protection (CNR-IRPI), Via Cavour 4/6, 87036 Rende, CS Italy; 2 Swedish Meteorological and Hydrological Institute (SMHI), Climate Information and Statistics, 601 76 Norrköping, Sweden; 3 National Research Council of Italy, Institute for Agriculture and Forest Systems in the Mediterranean (CNR-ISAFOM), Via Cavour 4/6, 87036 Rende, CS Italy

**Keywords:** Climate sciences, Hydrology

## Abstract

In order to correctly detect climate signals and discard possible instrumentation errors, establishing coherent data records has become increasingly relevant. However, since real measurements can be inhomogeneous, their use for assessing homogenization techniques is not directly possible, and the study of their performance must be done on homogeneous datasets subjected to controlled, artificial inhomogeneities. In this paper, considering two European temperature networks over the 1950–2005 period, up to 7 artificial breaks and an average of 107 missing data per station were introduced, in order to determine that mean square error, absolute bias and factor of exceedance can be meaningfully used to validate the best-performing homogenization technique. Three techniques were used, ACMANT and two versions of HOMER: the standard, automated setup mode and a manual setup. Results showed that the HOMER techniques performed better regarding the factor of exceedance, while ACMANT was best with regard to absolute error and root mean square error. Regardless of the technique used, it was also established that homogenization quality anti-correlated meaningfully to the number of breaks. On the other hand, as missing data are almost always replaced in the two HOMER techniques, only ACMANT performance is significantly, negatively affected by the amount of missing data.

## Introduction

Beside an increase in the frequency and intensity of extreme weather events, climate change can affect some major social and environmental factors, such as ecosystems, availability and quality of drinking water, agriculture and food production, economic development and migration, and for this reason in the last decades it has received an ever-growing attention^1^. In order to perform a reliable climate change analysis, good quality long-term time series are needed. In fact, if data are affected by quality issues, a consequence may be distorted and incorrect results^2^. In order to detect and eliminate errors in the data, a quality control investigation should be performed before analyzing observational records^3^. With this aim, the World Meteorological Organization (WMO) provides several documents with guidelines on quality control investigation^4–6^. Moreover, different quality control approaches have been presented in several research projects^2^. The problem of errors, uncertainty and validations is thus of major concern in the field of meteorological data homogenization. Usually, sources of uncertainty can be classified into two main types:

1. Biases, i.e., issues related to a specific measurement method; exposure issues (e.g., temperature recorded at land stations before the development of louvered screens); representativeness of the site in the context of possible land-use or environmental change at the measurement’s location or across the grid box within which it is located (e.g., effects of urbanization);
2. Inhomogeneities, i.e., issues related to changes in site location, times of day at which measurements are made, changes in methods to extract daily/monthly measures from measurements, changes in instrumentation^7,8^.

As regards the latter point, a climate time series is considered homogeneous when its variations are caused only by changes in weather and climate, thus without any influence of non-climatic factors that mislead the true climate variation^9^. Homogenization and missing value reconstruction of climate records have for a long time now been acknowledged as necessary steps for proper climatic analysis^10^. Homogenization of data records is attempted through the detection of inhomogeneities at annual, seasonal or monthly means level, and then adjusting the corresponding daily values through various techniques^11,12^. The detection of break points, where an inhomogeneity takes place (e.g., an instrument is replaced, an extraction method is changed, a ground station is moved), is of major importance in this process^13^. In the last years, many homogenization methods have been proposed and several works concerning the detection of inhomogeneities in long-term time series were performed all over the world^14^. For example, as regards temperature data, in a data reconstruction study for the Alpine region, Eccel et al.^15^ managed to decrease standard deviation of temperature in the range of 50%; this allowed to attribute at least 60% of the apparent climate signal to instrument inhomogeneities. In a homogenization study performed at the Poznan meteorological station in Poland, after a correction of 0.5–0.6 °C was applied, the temperature increase over 100 years was of about 1.1 °C^16^. The majority of the methods proposed in literature to detect and remove inhomogeneities in a series generally consider the relative homogenization approach. This approach is based on the assumption that the same climatic signal influences neighboring stations, and thus inhomogeneities can be identified considering the differences between these stations^17^. In relative homogeneity testing, the time series of the station being tested (candidate station) is compared to the ones of multiple surrounding stations (reference stations) either in a pairwise fashion or to a single composite reference time series computed from multiple neighboring stations^18^. Two well-known examples of homogeneous relative tests are the Standard Normal Homogeneity Test (SNHT^19^) and the Craddock test^20^.

In order to perform a quick analysis of the data, some authors prepared and documented ready-to-use computer packages based on the relative homogenization approach. Some of the most used packages are the ACMANT^21^, the ProClimDB^22^, the HOMER^23^, the MASH^24^ the RHtests^25^ and the USHCN^26^, that allow to detect and remove inhomogeneities of different climatic variables at different timescales. However, it is not possible to know whether the whole inhomogeneous part has been correctly attributed and totally removed from the climate signal. Moreover, another problem of homogenization procedures lies in how good the clean reference station or dataset used is^13^.

Just like other meteorological time series, homogenized datasets’ results must be assessed. In climatology and meteorology, validations are usually performed through comparison metrics between data that are considered real (for instance, ground stations data) and data to validate. For example, reanalysis precipitation is validated against existent pluviometric networks. These metrics are seldom general, but they need to be tailored upon the examined variable, the region of concern and the specific use of the validated data. Commonly used metrics include daily, seasonal and monthly comparisons of mean values, RMSE, and bias, correlation functions, probability distribution functions, efficiency coefficients like the Nash–Sutcliffe coefficient, etc. One of the main problems of homogenization is that its quantitative performance evaluation is difficult because true values are not generally known, i.e., there is no reference set that can be used for validation. An alternate procedure can be to validate homogenization techniques in order to establish the best one to use in a certain region for homogenizing a certain variable. This validation can be achieved through the creation of artificially inhomogeneous datasets, starting from coherent datasets, also called benchmark datasets. In turn, this can be done either by creating artificial datasets, or by using homogeneous datasets, i.e., sufficiently reliable ones, and then adding artificial inhomogeneities to the datasets. Establishing a benchmark dataset and defining evaluation metrics are a necessary step to any validation (e.g.^27^).

Within this context, this paper dealt with the following main questions:

(i) What metrics can be meaningfully used to validate the best-performing homogenization technique for a temperature record in a region?
(ii) Does temperature homogenization techniques’ performance depend on physical features of a station like its geographical position, i.e., latitude, altitude above sea level (a.s.l.) and distance from the sea?
(iii) Does temperature homogenization techniques’ performance depend on the nature of the inhomogeneities, i.e., the number of break points and missing data?

In order to answer these questions, in this study, two regional datasets of homogeneous maximum and minimum temperature records, one in southern Sweden and one in Slovenia, have been considered. The datasets have been artificially rendered inhomogeneous through the introduction of break points and missing data, and then homogenized through the use of three different techniques: ACMANT, HOMER, and a modified HOMER method.

The paper is thus organized as follows: The Methodology and Areas of study and data sections describe the homogenization techniques that were evaluated, the evaluation metrics used (bias, absolute error, root mean square error, Pearson correlation and factor of exceedance), and the regions and the temperature networks used for the evaluation, a 100-stations network in southern Sweden and a 30-stations network in Slovenia. These are followed by Results and discussion, where the evaluation results are presented along with the correlation analysis of station features (latitude, elevation and distance from the sea) and data characteristics (number of breaks, number of missing data) against evaluation metrics. The paper is finished by a Conclusions section.

## Methodology

Three homogenization techniques were evaluated in this study: ACMANT (Adapted Caussinus-Mestre Algorithm for Networks of Temperature series^21^), and two versions of HOMER (HOMogenization software in R^23^), the standard, automated setup mode (Standard-HOMER), and the manual mode setup (SMHI-HOMER) performed by the Swedish Meteorological and Hydrological Institute (SMHI).

Moreover, with the aim to attribute differences in performance of the homogenization techniques, the relationship between uncertainties and physical features of the stations or intrinsic characteristics of the inhomogeneities were analysed. The metrics of the stations of each regional dataset have been compared, through the use of Pearson correlation coefficients, to two different types of variables: physical features of the stations (latitude, distance from the sea, altitude) and features of the corrupted station data (the number of breaks and missing data introduced).

### ACMANT

ACMANT is a fully automatic homogenization tool based on the PRODIGE homogenization technique^28^. General features of ACMANT include homogenization through steps with increasing sensitivity, the detection of changes in seasonal cycles, and ensemble homogenization. ACMANT uses Automatic networking^29^ on deseasonalised data to find appropriate references for each time series. The selection of the networks is based on Spearman correlation and can vary from year to year. ACMANT fills data for each station taking into account start date and end date for records at each station. In the current study, ACMANT v4.3^30,31^ is used.

### HOMER

HOMER combines methods for detecting homogeneity breaks borrowed from PRODIGE^28^, ACMANT^21^, and a joint segmentation method originally developed for applications in genome research^32^. All the methods use maximum likelihood estimates to find optimal break points.

The correction is conducted with the ANOVA model^23,28^. HOMER has two modes of operation: an interactive and an automatic mode. In the interactive mode, HOMER suggests a number of possible break points identified by the different methods available. On the contrary, the automatic mode uses the breaks detected by the joint segmentation and the ACMANT methods but disregards the breaks detected by the PRODIGE one. In this study, BART, a recently developed script^33^, is used. BART can run HOMER automatically, either with a set that mimics the original automatic mode described above (standard-HOMER), or selecting automatically the PRODIGE seasonal and annual breaks, the ACMANT annual breaks and the joint-segmentation annual breaks (SMHI-HOMER).

Contrarily to ACMANT, HOMER tries to fill data in all series in a network searching for the earliest starting date and last ending date of the stations in the whole network.

### Evaluation metrics

In order to validate the results of homogenization techniques on climate data, some common evaluation metrics have been used. These metrics allowed to compare a real, homogeneous ground station dataset (*x*^*R*^) with a dataset *x*^*M*^ created through the homogenization of an artificially constructed inhomogeneous dataset pertaining to the same data, or obtained through a climate model or a reanalysis. Let N be the number of measurements. Then, some of the most commonly use devaluation metrics will be expressed in the following form:

(1) Bias (*B*)

1 $$ B = \frac{1}{N}\mathop \sum \limits_{j = 1}^{N} \left( {x_{j}^{M} - x_{j}^{R} } \right) $$

where *N* is the number of measured/modelled/homogenized value vs real value pairs $(x_{j}^{M} ,\;x_{j}^{R} )$. The bias can be positive or negative. Depending on its sign it shows average overestimation (+) or underestimation (−) of the measured/modelled/homogenized data compared to the clean ones. However, the bias does not provide any information regarding the number of overestimations or underestimations. A perfect model or homogenization algorithm would result in a 0 for this metric, though the $(x_{j}^{M} - x_{j}^{R} )$ values can be different from 0 even though their averaged sum is 0. In other words, $B = 0$ is a necessary, but not sufficient, condition for having a perfect model or algorithm.

(2) Absolute error (*B*^*abs*^)

2 $$ B^{abs} = \frac{1}{N}\mathop \sum \limits_{j = 1}^{N} \left| {x_{j}^{M} - x_{j}^{R} } \right| $$

Absolute error (sometimes called absolute bias) is used to provide an effective measure of the difference between the validated series and the validation set. In this case, *B*^*abs*^ = 0 is a necessary and sufficient condition for having a perfect model or algorithm. However, there is no information regarding the average sign of the difference (overestimation/underestimation).

(3) Factor of exceedance (*F*)

3 $$ F = \left( {\frac{{K_{{(x^{M} > x^{R} )}} }}{K} - 0.5} \right)100 $$

The factor of exceedance is a percentile measure that indicates how many values of the validated series exceed the corresponding values in the validation set. The term $K_{{(x^{M} > x^{R} )}}$ is the number of coupled values where an exceedance takes place. The factor goes from *F* = − 50% (all the values of the validated series are underestimations of the reference set) to *F* = + 50% (all the values of the validated series are overestimations of the reference set). If *F* = 0% then the sets are identical (perfect modelling/homogenization/measurement).

(4) Root Mean Squared Error (*RMSE*)

4 $$ RMSE = \sqrt {\frac{{\mathop \sum \nolimits_{j = 1}^{N} (x_{j}^{M} - x_{j}^{R} )^{2} }}{N}} $$

The Root Mean Square Error (*RMSE*) is defined as the square root of the mean squared error, i.e., the average squared difference between the estimated values and the actual value. In this case, each difference term is replaced by the difference between the validated value and the corresponding value in the validation set.

*RMSE* provides us with information about an average deviation of the validated data from the validation set but, like all metrics that are absolute values, it does not provide information about over- or under-estimation. There is no intrinsic upper limit to the *RMSE*, but huge errors (say, several K or more for temperature) can indicate a broken instrument, an inadequate model or a wrong technique is being used.

(5) Pearson’s correlation coefficient (*CC*)

5 $$ CC = \frac{{\mathop \sum \nolimits_{j = 1}^{N} \left( {x_{j}^{M} - \overline{x}^{M} } \right)\left( {x_{j}^{R} - \overline{x}^{R} } \right)}}{{\sqrt {\mathop \sum \nolimits_{j = 1}^{N} \left( {x_{j}^{M} - \overline{x}^{M} } \right)^{2} \mathop \sum \nolimits_{j = 1}^{{N_{k} }} \left( {x_{j}^{R} - \overline{x}^{R} } \right)^{2} } }} $$

In the equation, $\overline{x}^{M}$ and $\overline{x}^{R}$ are calculated as:

6 $$ \overline{x}^{M} = \frac{1}{N}\mathop \sum \limits_{j = 1}^{N} x_{j}^{M} $$

7 $$ \overline{x}^{R} = \frac{1}{N}\mathop \sum \limits_{j = 1}^{N} x_{j}^{R} $$

Pearson correlation coefficient is a measure of the linear relationship between two variables or datasets. *CC* can vary from − 1 (perfect anticorrelation or negative correlation) and + 1 (perfect correlation or positive correlation), while 0 means there is no correlation at all. In this study, the best result is the perfect positive correlation between the validation set and the validated dataset, i.e. *CC* = 1. In literature, generally values from 0.4 to 0.6 are said to yield weak correlation, values from 0.6 to 0.8 to yield correlation, and values above 0.8 to yield strong correlation.

## Areas of study and data

### Southern Sweden

**Figure 1:**
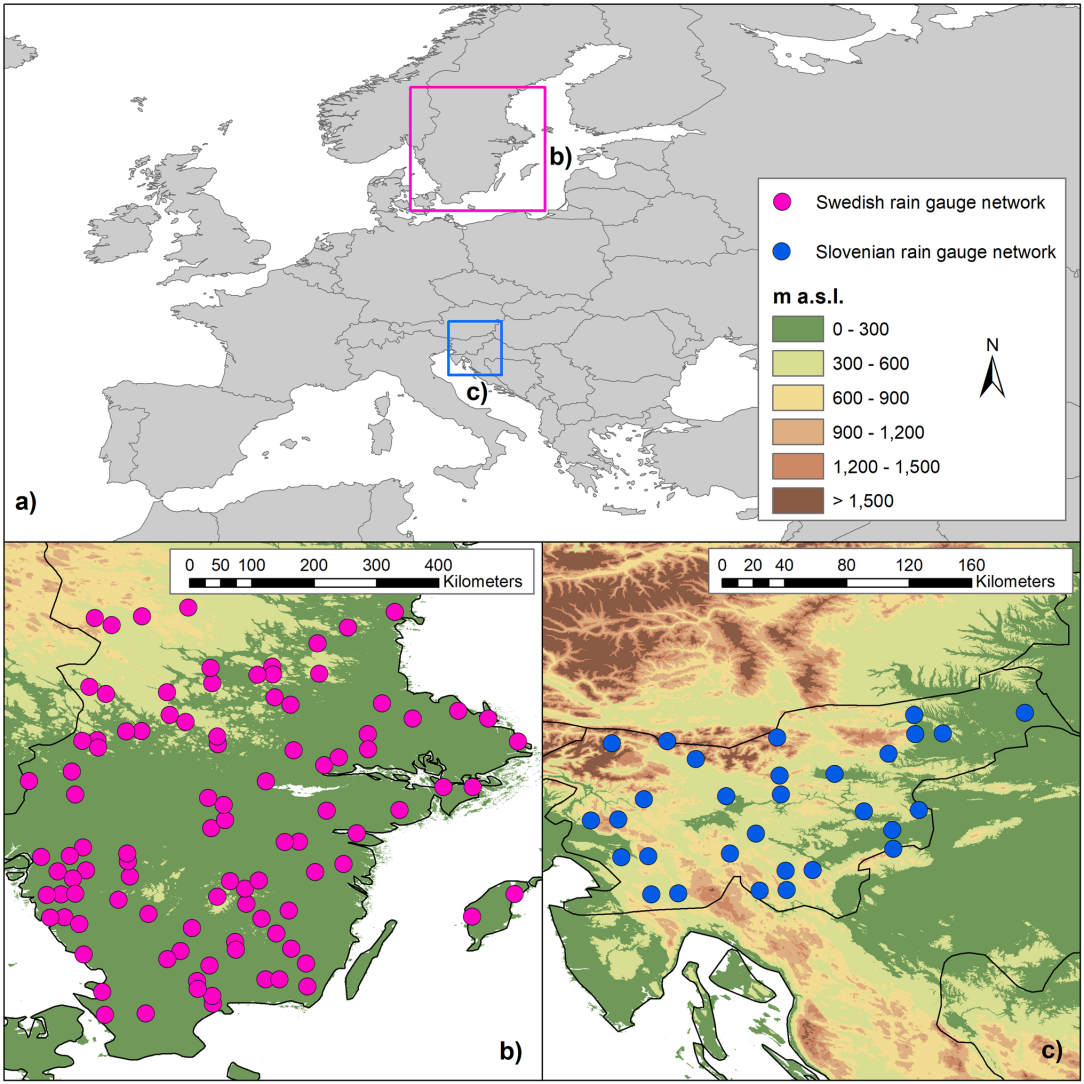
Localization of the study areas (**a**) and of the stations on a Digital Elevation Model (DEM) of southern Sweden (**b**) and Slovenia (**c**).

The climate in Southern Sweden (Fig. 1) is dominated by mild and humid south-westerly winds from the Atlantic Ocean. The maritime climate is characterized by cool summers and mild winters, especially along the west coast. In the central part of the region, a highland area, with the altitude of around 200–300 m above sea level, has a slightly more continental climate. The annual average temperature for the normal period 1961–1990 is 6–7 °C along the coast and 5–6 °C in the highland. The annual precipitation is highest in the western part of the region adding up to about 1000 mm. The lowest annual precipitation is found in the south east part of the region with 500 mm^34^.

### Slovenia

Slovenia is a European country located at the transition of the Alps to the Dinaric range and at the transition of the Mediterranean to the Pannonian Basin (Fig. 1). Its transitional climate is driven by the interaction between maritime and continental masses, with local conditions strongly influenced by the diversity in terrain and altitude^35^.

Three climate types characterize this region, according to the Köppen–Geiger classification^36^: the temperate humid climate with hot summers, the temperate humid climate with warm summers, and the mountainous climate. Mean annual air temperature presents a high spatial variability, dropping from 15 °C in south‐western part to below 0 °C in north‐western part of the county^37^.

### Data

To perform the study, a region with a network of high-quality, quasi-homogeneous station temperature datasets with long time series was needed. Two such regional networks were readily available from the INDECIS Project data repository to use: one consisting of 100 ground stations in Sweden (Fig. 1b), the other of 30 stations in Slovenia (Fig. 1c), compiled from reanalysis data performed by KNMI^38,39^. Both datasets regard maximum (T_max_) and minimum monthly (T_min_) temperature at the monthly scale for the 1950–2005 time period. The inhomogeneous datasets were created through the introduction of artificial breaks and missing data, with a different setup for each station. There can be 0 to 7 breaks both in Slovenia and southern Sweden stations for both variables; missing data for southern Sweden stations are up to 256 (with 111 on average) for T_max_ and up to 250 (with 104 on average) for T_min_; while for Slovenia stations they are up to 229 (with an average of 98) for T_max_, and up to 214 (with an average of 117) for T_min_.

**Figure 2:**
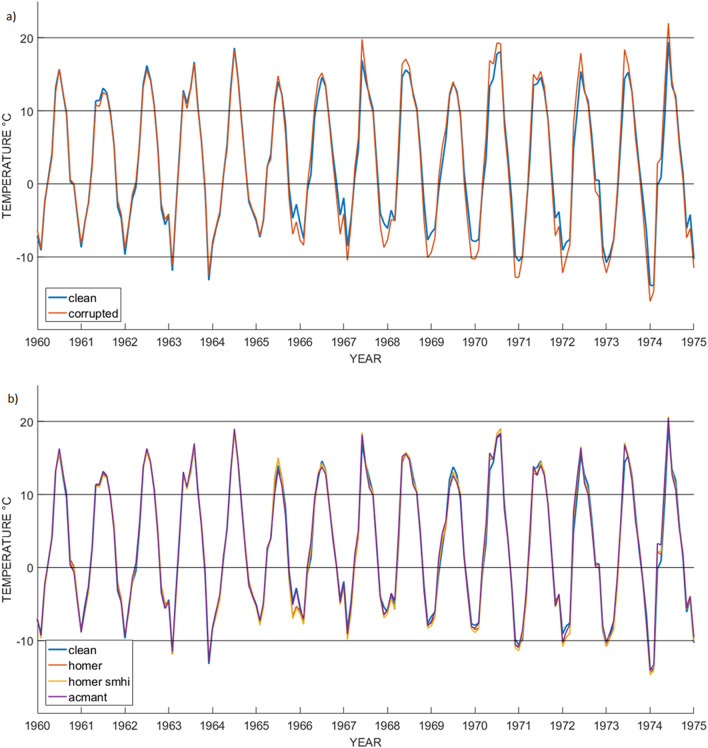
Maximum temperature sample for station no. 98 in the S Sweden network, for years 1960–1975. (**a**) Original clean data vs corrupted data; (**b**) Original clean data vs three different homogenized data.

At this stage, the three homogenization techniques were applied to each artificially inhomogeneous (i.e., corrupted) set. A sample from the corruption and homogenization of one of the stations (maximum temperature, from the Swedish dataset), is shown in Fig. 2. In order to perform the validation, the mean values of the metrics for the whole dataset have been calculated. Given a generic metric *M*, where *M* can be *B*, *Babs*, *RMSE*, *CC*, or *F*, then the regional mean metric is:

8 $$ \overline{M} = \frac{{\mathop \sum \nolimits_{s = 1}^{S} (M_{s} )}}{S} $$

where *S* is the number of ground stations in the regional dataset (*S* = 100 for southern Sweden, *S* = 30 for Slovenia), while the small $s$ refers to each individual station.

The same metrics were also applied to study the differences and errors between the inhomogeneous datasets and the clean data; in this way, it was possible to evaluate quantitatively the improvements obtained by the use of homogenization techniques.

## Results and discussion

Tables 1 and 2 show the results of the regional mean metrics calculated for the corrupted dataset and the three homogenized datasets, for southern Sweden and Slovenia respectively. Results are considered significant at the 95% statistical level.

**Table 1:** 100-station average of five metrics of corrupted dataset and homogenization techniques for Southern Sweden compared to the clean data.

Method	Variable	Corrupted	Standard HOMER	SMHI HOMER	ACMANT
RMSE	T_max_	0.71	0.50	0.51	0.46
	T_min_	0.89	0.60	0.61	0.53
B	T_max_	− 0.03	− 0.03	− 0.04	− 0.01
	T_min_	− 0.04	0.00	− 0.03	− 0.01
B^abs^	T_max_	0.38	0.30	0.31	0.25
	T_min_	0.48	0.36	0.36	0.28
F	T_max_	− 28.4%	− 21.4%	− 22.2%	− 23.1%
	T_min_	− 29.0%	− 19.5%	− 22.6%	− 23.9%

**Table 2:** 30-station average of five metrics of corrupted dataset and homogenization techniques for Slovenia compared to the clean data.

Method	Variable	Corrupted	Standard HOMER	SMHI HOMER	ACMANT
RMSE	T_max_	2.14	0.80	0.79	0.77
	T_min_	1.31	0.87	0.83	0.76
B	T_max_	0.14	0.15	0.01	0.02
	T_min_	0.09	0.05	0.02	− 0.03
B^abs^	T_max_	1.34	0.49	0.48	0.42
	T_min_	0.67	0.48	0.48	0.37
F	T_max_	− 21.3%	− 7.3%	− 14.0%	− 20.3%
	T_min_	− 29.3%	− 19.7%	− 20.1%	− 28.4%

It is clear from the metrics results that homogenization improves the correspondence of the dataset to the real data on all accounts except for the bias. *RMSE*, *B*^*abs*^ and *F* all allow to evaluate quantitatively meaningful improvements in the homogenized datasets. For instance, for maximum temperature, mean *RMSE* in Sweden is reduced from 0.71 (corrupted dataset) to 0.50 (standard HOMER), 0.51 (SMHI-HOMER) and 0.46 (ACMANT); for minimum temperature, mean RMSE was reduced from 0.9 to 0.6, 0.61 and 0.53 respectively. In both cases, there was an improvement of about 30% with respect to the corrupted dataset. In Slovenia, improvements are even bigger in absolute terms, as the Slovenian corrupted dataset has much worse *RMSE* to start with: *RMSE* is 2.1 for maximum temperature and 1.31 for minimum temperature, while the homogenized *RMSEs* are respectively 0.8 and 0.87 (standard HOMER), 0.79 and 0.83 (SMHI-HOMER), 0.77 and 0.76 (ACMANT).

However, the Pearson correlation coefficient (not shown) didn’t prove a good metric to assess the quality of the process. First of all, it didn’t improve much in either region or for either variable. The reason is that, even though artificially manipulated, the corrupted data still show a very high linear correlation with the real one, as is expected in the case of inhomogeneities to the instrumental sensitivity or the re-positioning of an instrument that are simulated by the introduction of artificial break points. For what concerns to the bias, it is true that homogenization not always improves this metric: for example, the southern Swedish maximum temperature corrupted dataset has a mean bias of − 0.03, while standard HOMER’s bias is − 0.03 and SMHI-HOMER’s − 0.04. However, taking into account both the bias and the absolute error (which shows reductions from 0.38 to 0.30 and 0.31 respectively), it is clear that the biases in the maximum temperature cancel out and the value goes towards zero, but this masks the true signal of the error. Validating the techniques through the bias, thus, can be used to assess if homogenization changes the sign of the bias, but it is not really suggested as a way to assess quantitatively whether there are improvements in the quality of the data.

According to the results, ACMANT is the best performer with regard to *RMSE* and absolute error for both regions and both variables; in these instances, the two HOMER techniques are almost equivalent, with very small differences for both of these metrics. For what regards the factor of exceedance, the two HOMER techniques perform best, with standard HOMER being slightly the better one (T_max_ Sweden: − 21.4% vs − 22.2%;T_min_ Sweden: − 19.5% vs − 22.6%; T_max_ Slovenia: − 7.3% vs − 14.0%; T_min_ Slovenia − 19.7% vs − 20.1%).

Comparing results in southern Sweden and Slovenia, it is also clear that the homogenization produces different outcomes depending on the variable and on the region. For example, while in Sweden the factor of exceedance (*F*) goes from the − 28.4% (for the corrupted dataset T_max_) and − 29.0% (for the corrupted dataset T_min_) to − 21.4% and − 19.5% respectively for the homogenized values T_max_ and T_min_ by means of standard HOMER, in Slovenia the factor of exceedance is − 21.3% and − 29.3%, for the corrupted dataset T_max_ and T_min_ respectively, and − 7.3% and − 19.7%, for the corresponding homogenized values with standard HOMER. That is to say that in Slovenia the homogenization improves much more than in southern Sweden according to this metric.

**Figure 3:**
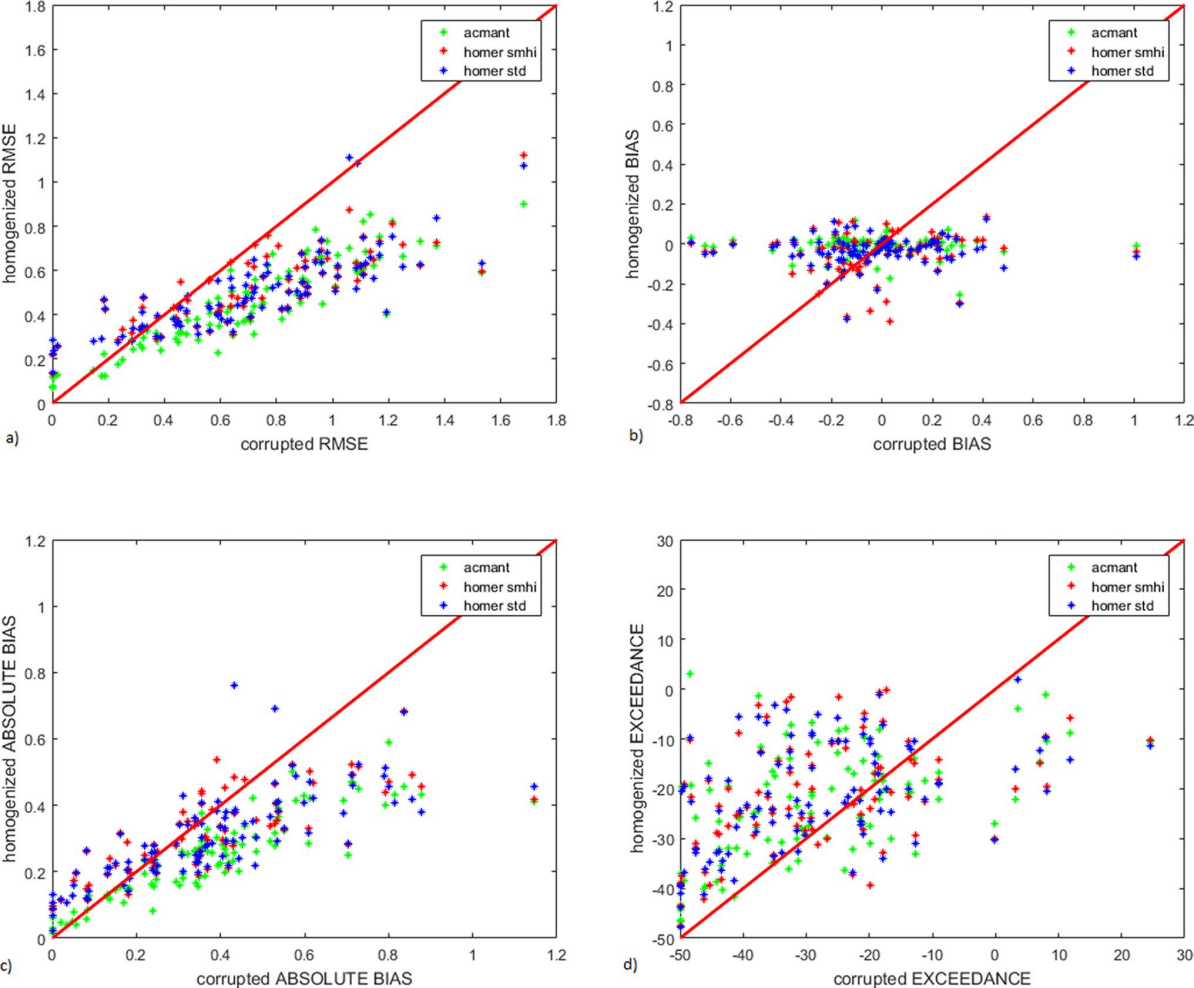
Sample statistics for metrics of the individual stations in the S Sweden dataset. Comparisons are between corrupted data and homogenized data for (**a**) RMSE; (**b**) bias; (**c**) absolute bias; (**d**) factor of exceedance. Comparison of RMSE, factor of exceedance, bias and absolute bias in corrupted data vs homogenized data for maximum temperature in the S Sweden database.

In Fig. 3, a sample breakdown of the metrics for the individual stations is shown; in particular, the sample for maximum temperature in Southern Sweden. The improvements from Table 1 are well represented for RMSE (where stations below the red diagonal indicate an improvement of homogenized data RMSE compared to corrupted data), absolute bias (stations under the red diagonal) and factor of exceedance (stations above the red diagonal). No significant pattern emerges from the bias, proving once again the little usefulness of this metric to evaluate homogenization results.

### Latitude

**Figure 4:**
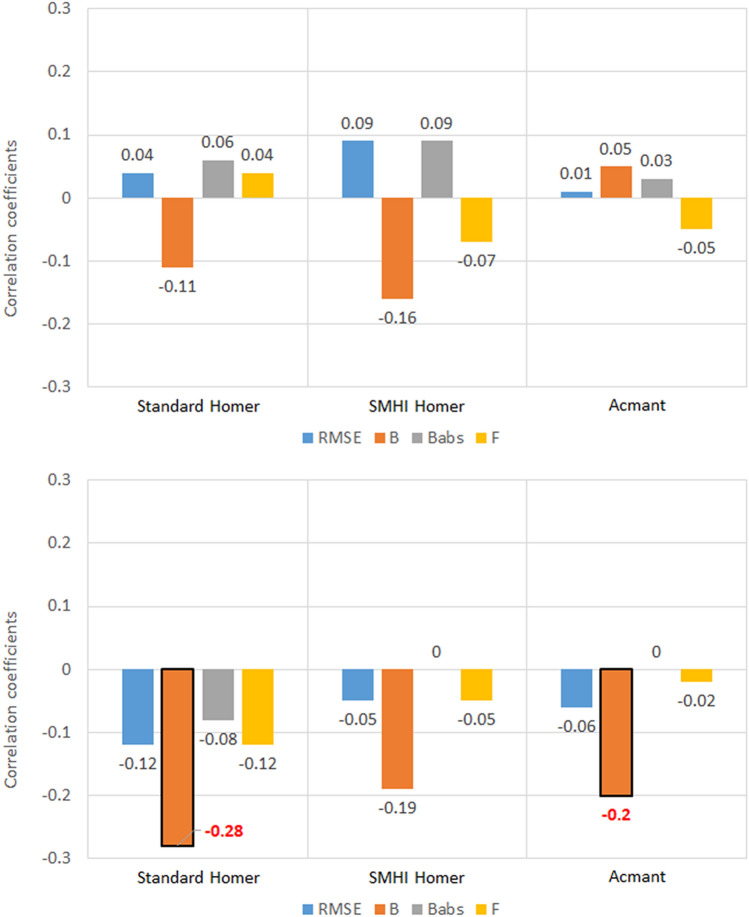
Correlation coefficients between the five metrics of homogenized temperature datasets and the station latitude, for the 100 southern Sweden stations. Values highlighted in red are at least 95% significant.

As regards southern Sweden, a huge difference in the values of the five metrics for each station belonging to the two regional sets was detected (Fig. 4).

No significant correlations were found between the latitude of the stations and the five metrics for T_max_. The weak correlations of the Bias metric (*B*) with latitude are also not relevant, considering the diminished role of the *B* metric determined in the analysis above.

**Figure 5:**
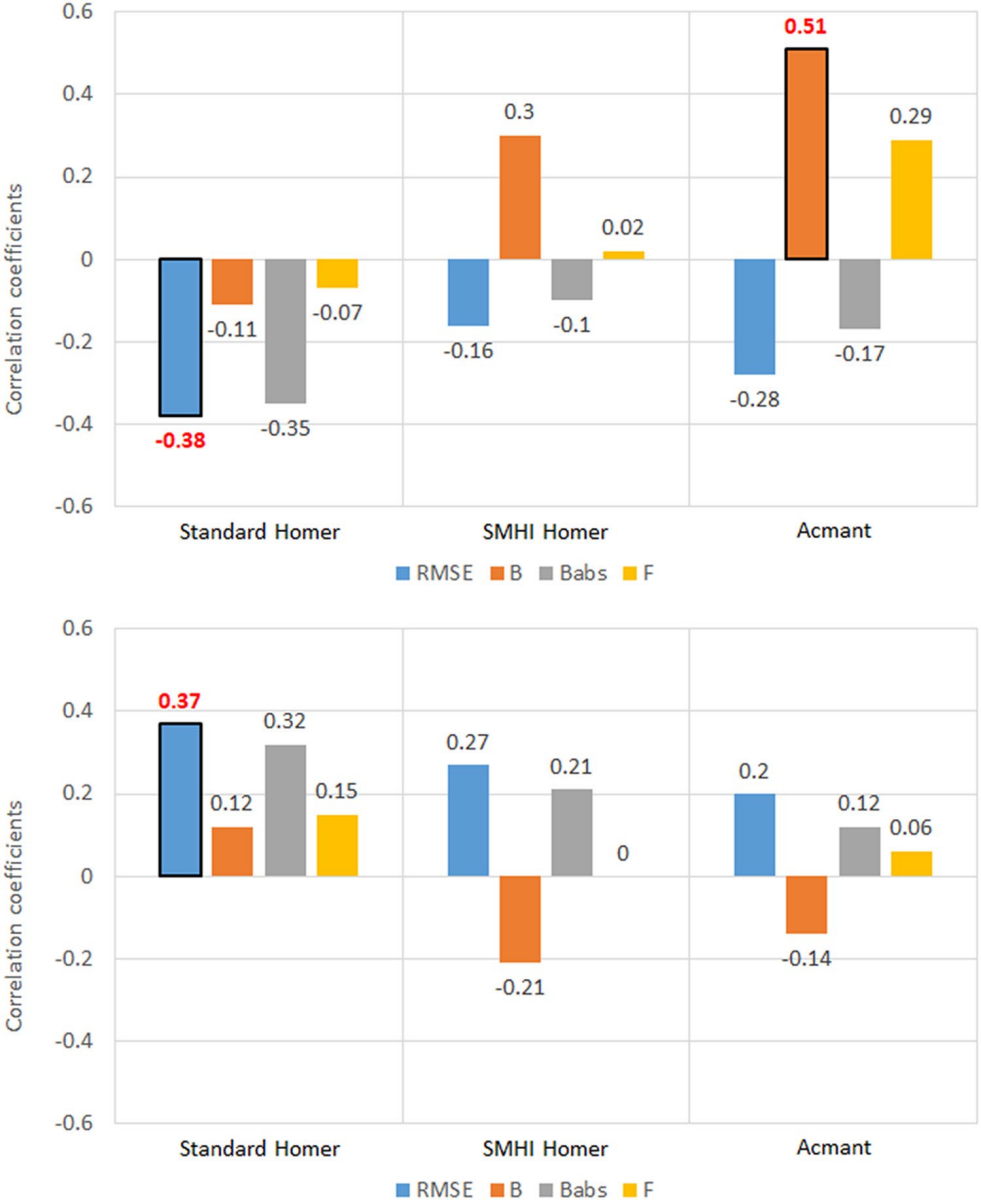
Correlation coefficients between the five metrics of homogenized temperature datasets and the station latitude, for the 30 Slovenia stations. Values highlighted in red are at least 95% significant.

For what regards Slovenia (Fig. 5), the only relevant results seem to be those for RMSE in Standard HOMER, where *CC* = − 0.38 for T_max_ and CC = 0.37 for T_min_ have been found. As the difference in latitude range is much smaller than in Sweden, it is difficult to establish whether this is truly a standout result. In general, there seems to be no major discernible pattern in the correlation coefficients depending on variable (T_max_ or T_min_) or region (southern Sweden or Slovenia).

### Distance from the sea

**Figure 6:**
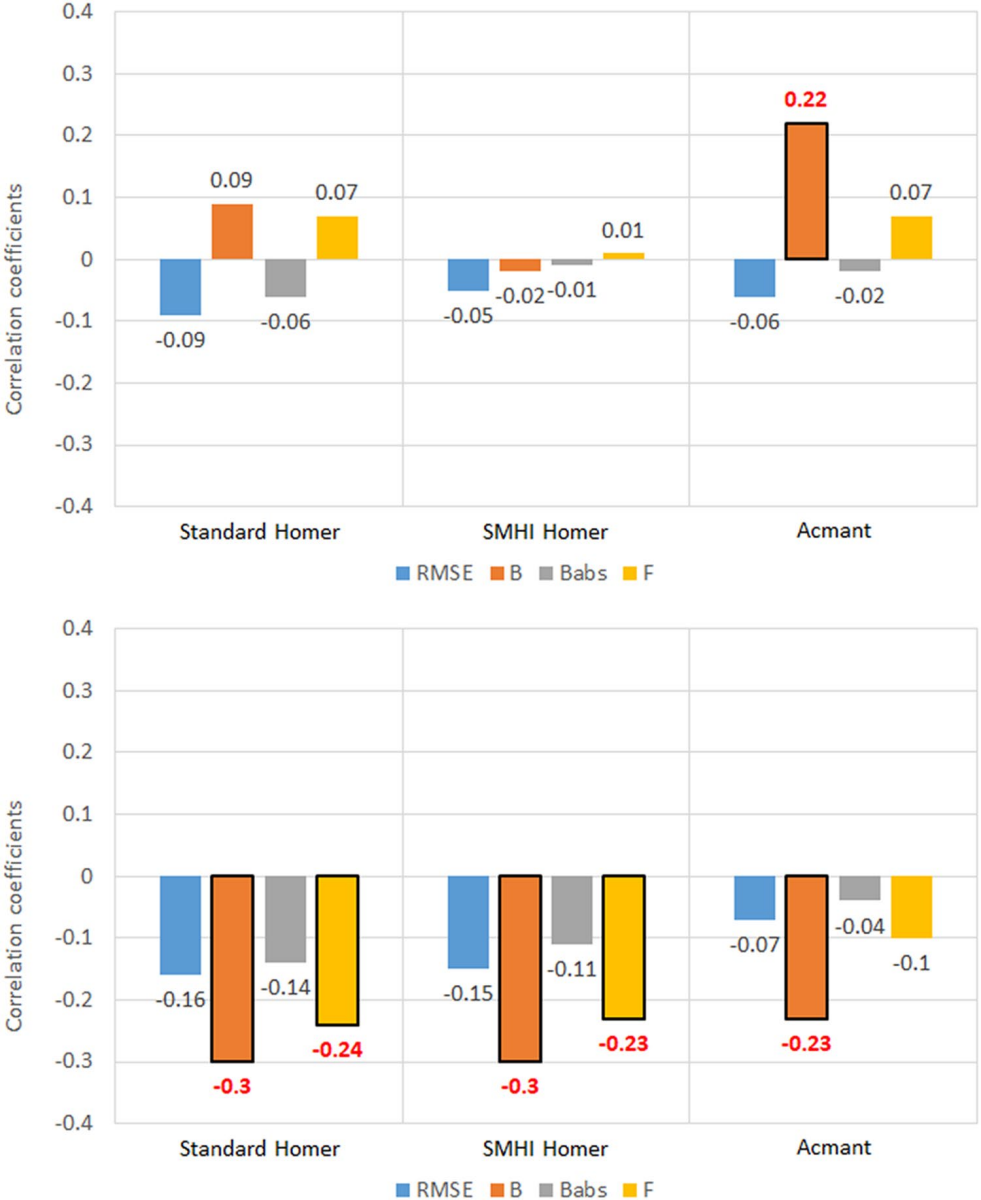
Correlation coefficients between the five metrics of homogenized temperature datasets and the station distance from the sea, for the 100 southern Sweden stations. Values highlighted in red are at least 95% significant.

Very weak correlations were found between the distance from the sea of the Swedish stations and the five metrics (Fig. 6). The weak correlations of the Bias metric (*B*) with distance from the sea are also not relevant, considering the diminished role of the *B* metric determined in the analysis above. On the other hand, the weak but significant negative correlation between Factor of exceedance (F) and the minimum temperature in Sweden for the HOMER homogenization techniques is more interesting. In fact, CC values of − 0.24 and − 0.23 have been detected for the standard HOMER and the SMHI HOMER, respectively. As all three methods were found to underestimate the values of the validated series, from these results it seems that increasing the station distance from the sea, the number of underestimated data increases slightly as well.

### Station altitude (a.s.l.)

**Figure 7:**
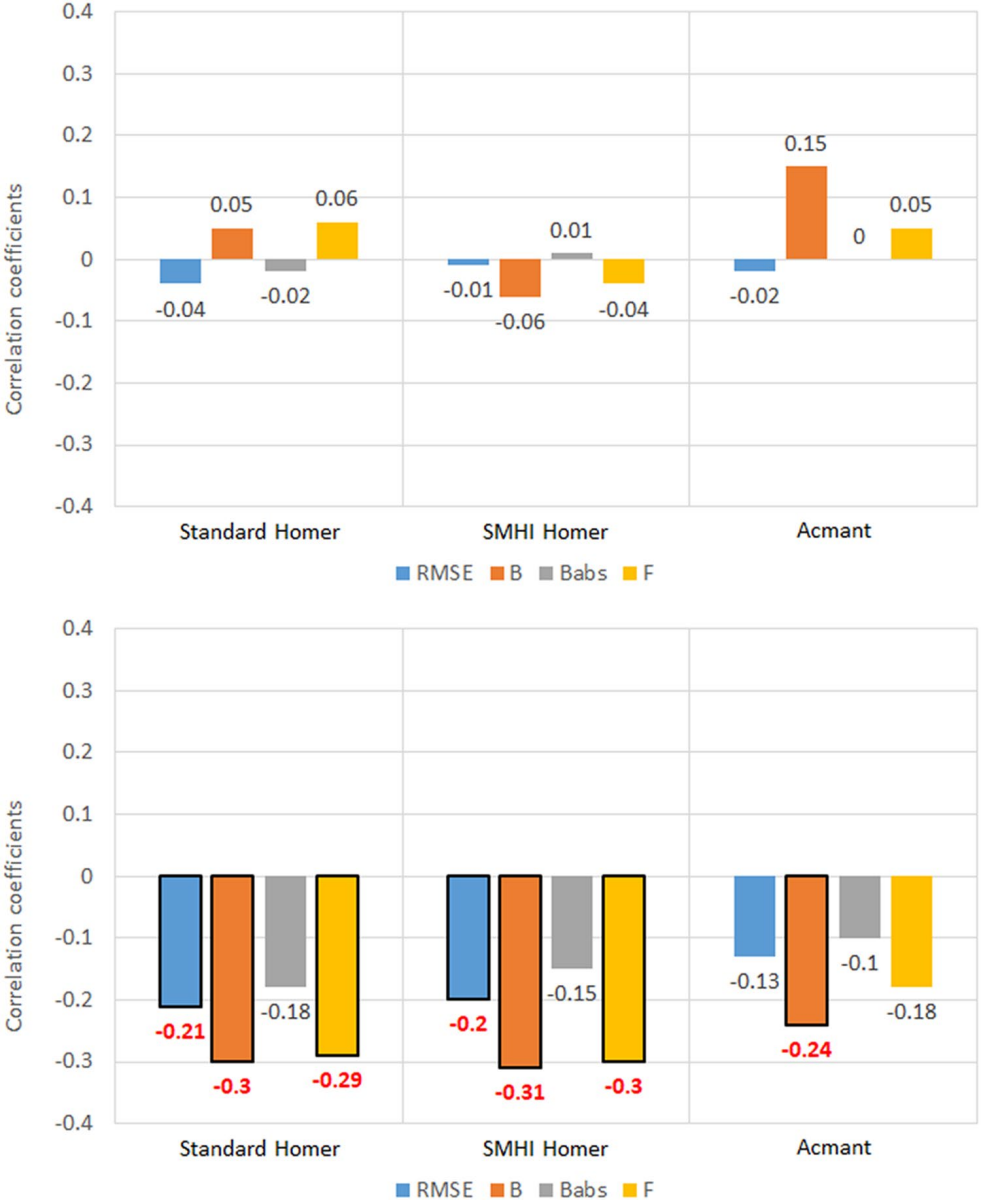
Correlation coefficients between the five metrics of homogenized temperature datasets and the station altitude, for the 100 southern Sweden stations. Values highlighted in red are at least 95% significant.

Like in the case of latitude, no significant correlations were found between station altitude and homogenization metrics for the maximum temperature (Fig. 7). On the other hand, a signal emerged linking altitude and minimum temperature for factor of exceedance (− 0.29 for standard HOMER and − 0.30 for SMHI HOMER). In particular, the negative correlations in *RMSE* and *F* that show up in the HOMER homogenizations of minimum temperature suggest that the temperature at stations with higher altitude might be slightly underestimated than that of stations at lower altitude. Since this effect is weaker both in magnitude and significance in ACMANT homogenization results, this might indicate that the latter technique is more apt to correctly infer minimum temperature data in southern Sweden.

### Number of breaks

**Figure 8:**
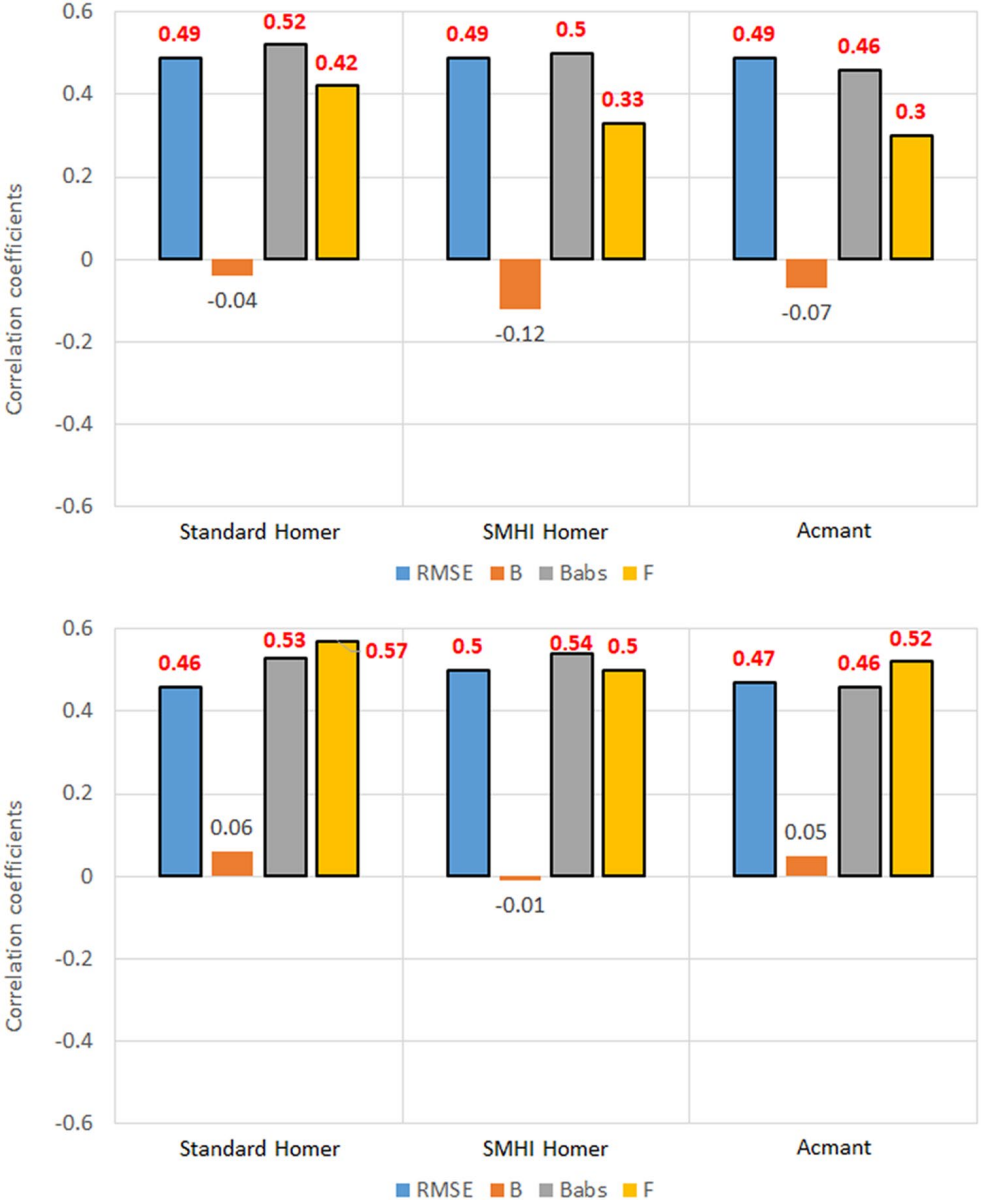
Correlation coefficients between the five metrics of homogenized temperature datasets and the number of breaks introduced in the corrupted set, for the 100 southern Sweden stations. Values highlighted in red are at least 95% significant.

**Figure 9:**
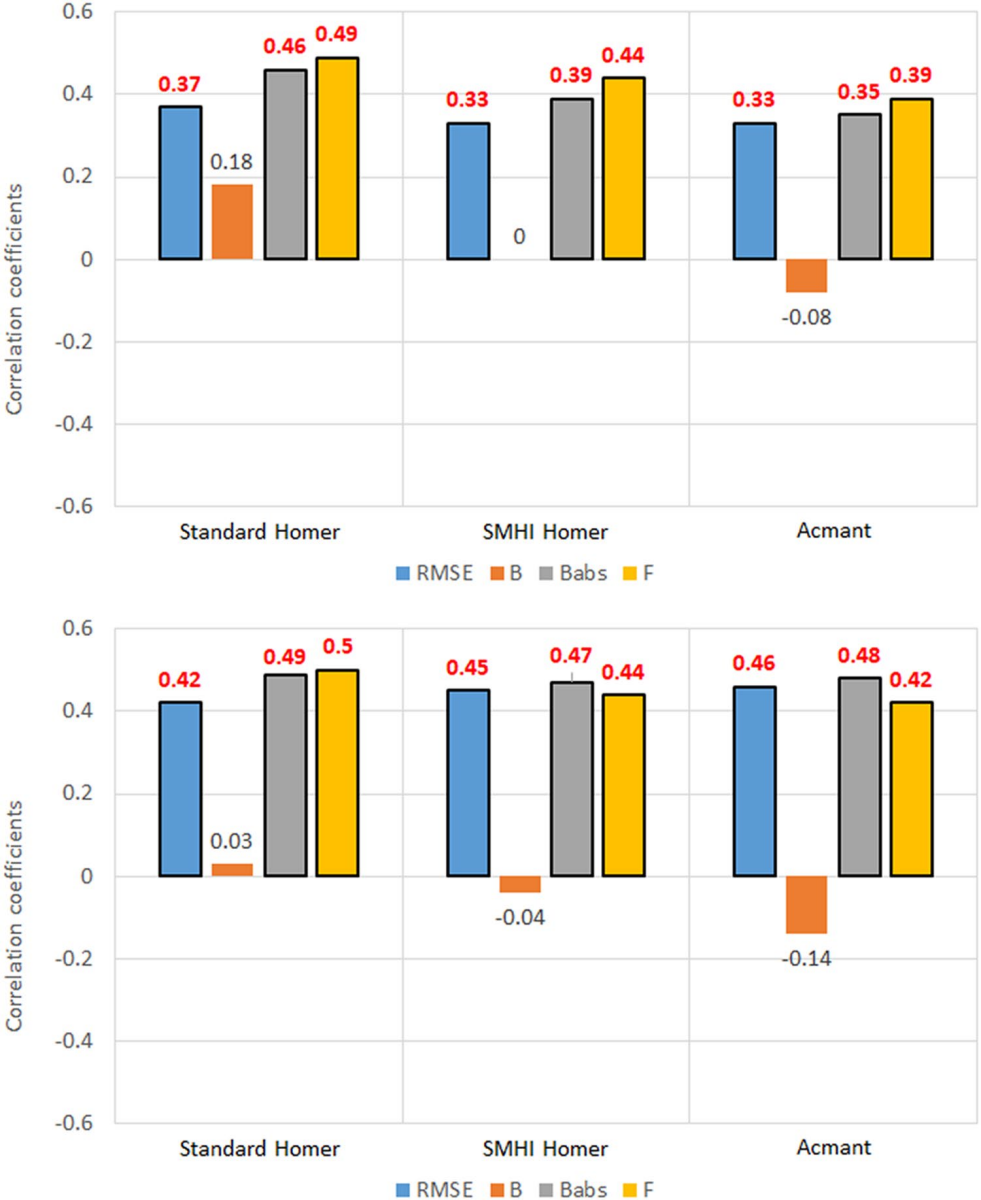
Correlation coefficients between the five metrics of homogenized temperature datasets and the number of breaks introduced in the corrupted set, for the 30 Slovenia stations. Values highlighted in red are at least 95% significant.

Results show that there is a moderate correlation between the number of breaks and the skill of the homogenization techniques (Figs. 8, 9).

It must be noted that in all these instances, the best performing technique will be the one where the relationship is least relevant. In the case of *RMSE* and *B*^*abs*^, a positive correlation means that the magnitude of errors increases with the number of breaks. In the case of the exceedance factor, a strong correlation, whether negative or positive, will mean that with the increase of break points, underestimation or overestimation increase too, respectively.

Last but not least, the bias does not show any correlation: this is probably related to the intrinsic nature of the bias metric, as it is not adjusted for magnitude like the absolute error. Absolute error results prove that there is in fact a correlation between bias and number of breaks, but that correlation does not show when the sign of the bias is not accounted for.

The metric that shows the strongest correlation with the number of breaks is the exceedance factor *F*, ranging from 0.30 (ACMANT maximum temperature in southern Sweden) to 0.57 (standard HOMER minimum temperature in Slovenia).

There are some slight differences between the metrics in southern Sweden (Fig. 8) and Slovenia (Fig. 9) in both variables: as the results for maximum temperature in Sweden are greater in magnitude and more robust statistically for *RMSE* and *B*^*abs*^, probably the different size of the sampling (100 stations against 30) means that the correlation is highlighted as the number of stations in the regional dataset increase. On the other hand, the correlation between maximum temperature exceedance factor and the number of breaks is stronger in the Slovenian case than in the southern Swedish one (in Slovenia 0.49 for standard HOMER, 0.44 for SMHI-HOMER and 0.39 for ACMANT, versus 0.42, 0.33 and 0.30 respectively in southern Sweden), so maybe in the former instance the correlation might be overestimated, again because of the difference of sampling size.

On the other hand, there are some differences between maximum temperature homogenization and minimum temperature homogenization. While in the case of RMSE and B^abs^ the increase in number of stations from Slovenia to Sweden results once again in stronger correlation, i.e., the increase in number of breaks yields worse results, this also happens for F, contrarily to the maximum temperature case. Moreover, the correlation between homogenized minimum temperature and the clean dataset is less influenced by the number of breaks in southern Sweden (− 0.31, − 0.34 and − 0.29) than in Slovenia (− 0.39, − 0.40 and − 0.38), contrarily to results with maximum temperature.

**Figure 10:**
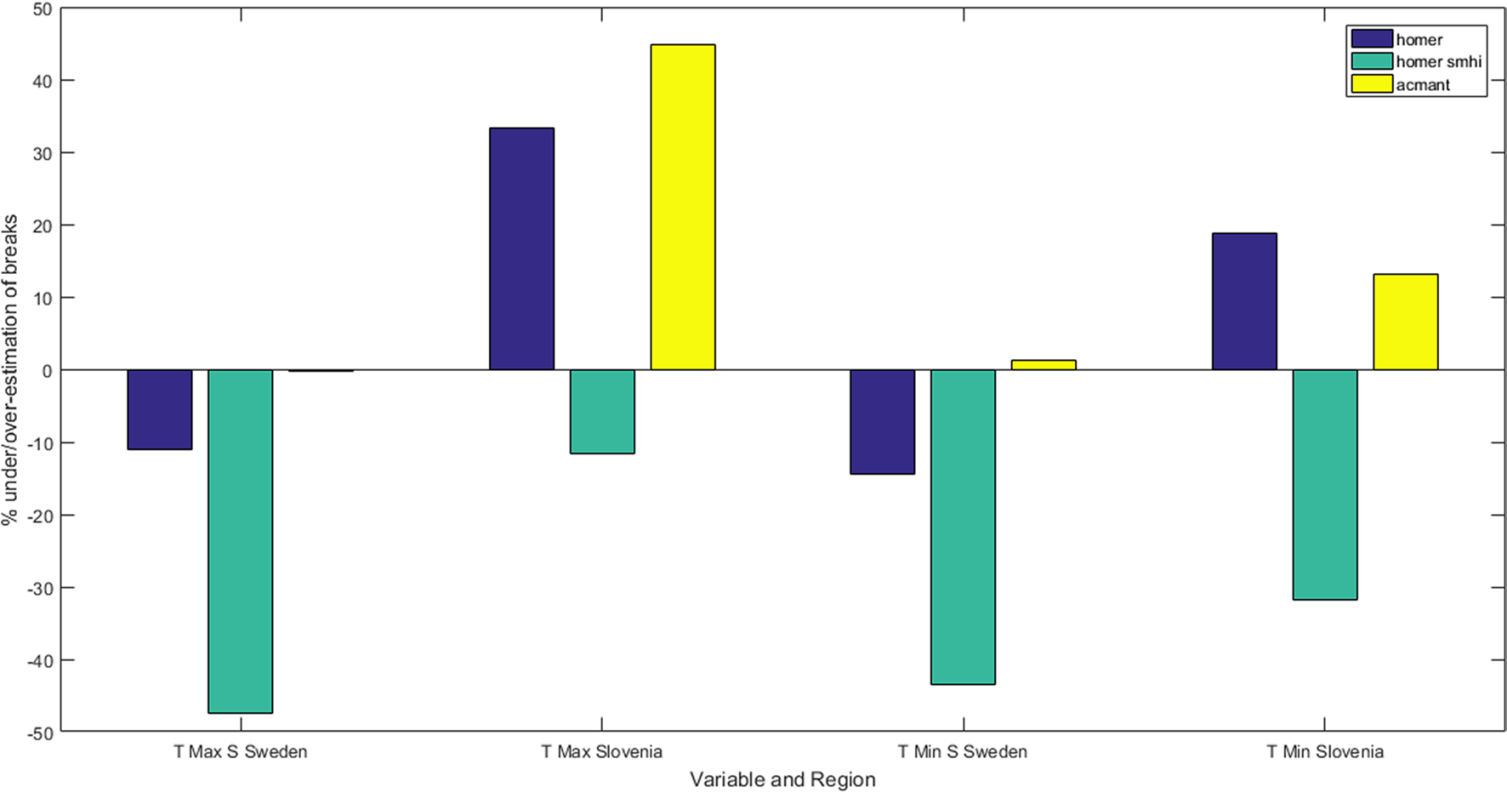
Percentage of detected breaks compared to true breaks, i.e., artificial breaks introduced in the series, using the three homogenization methods (HOMER, SMHI-HOMER and ACMANT), for T_max_ and T_min_ in Sweden and Slovenia. Positive percentages indicate overestimation of breaks, while negative percentages indicate underestimation of breaks.

It is possible to conclude that a higher number of break detections generally lowers the error, but it might be justified to use some caution when handling real data without any validation data set. This becomes clear by looking at the percentage of estimation of true breaks in the homogenization, calculated and shown in Fig. 10. Here, negative percentages indicate an underestimation of breaks, i.e., less breaks than true ones were detected; while positive percentages indicate overestimation, i.e., more breaks were detected than there really are. From the figure, it is possible to see that while standard-HOMER and ACMANT perform better in evaluating breaks than SMHI-HOMER for Southern Sweden (maximum temperature: 249, 279 and 149 breaks respectively compared to 280 true breaks; minimum temperature: 221, 261 and 146 breaks respectively compared to 258 true ones), this is not true of Slovenia, especially for the maximum temperature scenario (104, 113 and 69 respectively compared to 78 true breaks).

### Missing data

With regard to missing data, it is important to note that HOMER and ACMANT have different approaches. HOMER fills-in missing data much more drastically than ACMANT (see Table 3): for instance, for maximum temperature, there are on average 111 missing data in southern Sweden stations and 98 missing data in Slovenia stations. These missing data are completely replaced in the HOMER homogenization, while with ACMANT 63 and 53 missing data respectively remain on average per station.

**Table 3:** Missing data in South Sweden (S Sweden) and Slovenia corrupted and homogenized datasets: maximum and mean number of missing data in the stations set.

	Corrupted dataset		Standard HOMER			SMHI HOMER			ACMANT		
	S Sweden	Slovenia	S Sweden		Slovenia	S Sweden		Slovenia	S Sweden		Slovenia
**T**_**max**_											
Max	256	229	19		9	19		9	167		156
Mean	111	98	0		0	0		0	63		53
**T**_**min**_											
Max	250	214	97		28	97		28	168		168
Mean	104	117	2		4	2		4	63		71

**Figure 11:**
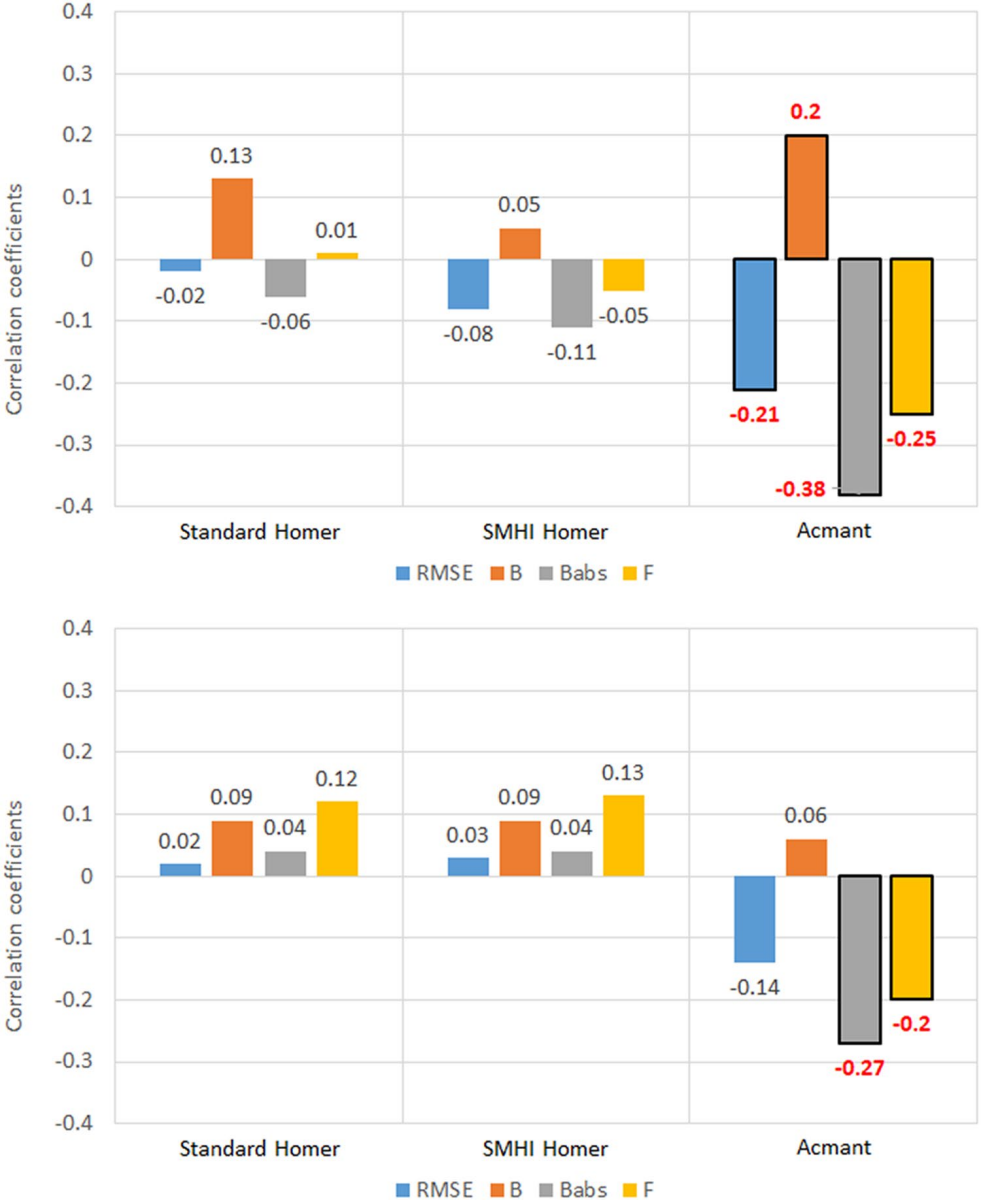
Correlation coefficients between the five metrics of homogenized temperature datasets and the number of missing data introduced in the corrupted set, for the 100 southern Sweden stations. Boxes highlighted in grey mean the value is at least 95% significant.

**Figure 12:**
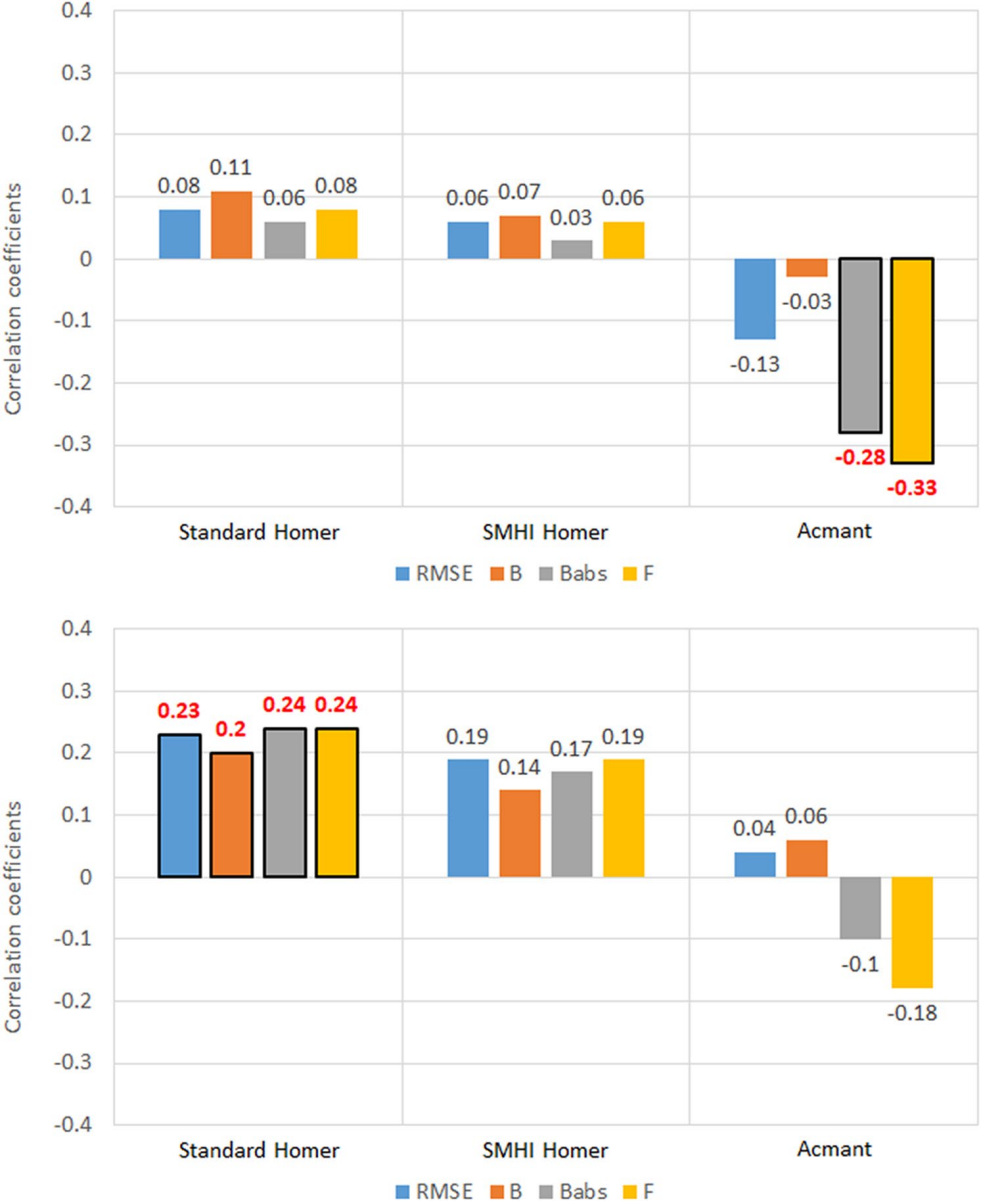
Correlation coefficients between the five metrics of homogenized temperature datasets and the number of missing data introduced in the corrupted set, for the 30 Slovenia stations. Boxes highlighted in grey mean the value is at least 95% significant.

Figures 11 and 12 show the results of the correlation between the missing data for each station and each metric used in this study. Since HOMER replaces the missing values almost entirely, it is clear that, as can be expected, the number of missing data is not significant for the metrics. It might be that, with much more missing data, the skill of the homogenization method to repair the dataset could break down, but it might happen at a number of missing data so big to make the actual dataset de facto useless.

On the other hand, for what regards the ACMANT technique, since much fewer missing data are replaced, the number of missing data bears an impact on the skill of the homogenization. Especially *B*^*abs*^ and *F* are significantly affected, for both regional datasets in both variables.

The one exception to this pattern happens for minimum temperature in Slovenia. Here, there is no significant relationship between missing values and ACMANT performance, while some significant, albeit weak, correlation between HOMER and missing data have been identified, especially for the Standard-HOMER technique. It is very difficult to pinpoint to an explanation for this difference, as the magnitude in the number of missing data remains the same.

## Conclusions

The main aim of this study was to develop a general method to validate homogenization techniques for a temperature record in a region. In order to determine which common metrics could provide meaningful results, an evaluation of the skill of various homogenization techniques in detecting and correcting inhomogeneities in monthly temperature data for two regional datasets (southern Sweden and Slovenia, made up of 100 and 30 climatological stations respectively) was conducted for 1950–2005. Techniques used were ACMANT and two different HOMER setups (the standard setup and a customized setup). It is important to note that these techniques were used as their homogenization results were readily available for Sweden and Slovenia through the INDECIS Project, but that the study can be replicated for any possible set of homogenization techniques. Maximum and minimum temperature were used as test variables. The skill was tested through the use of five evaluation parameters: Pearson correlation, root mean square error (*RMSE*), bias, absolute error and factor of exceedance. Results showed that *RMSE*, absolute error and factor of exceedance are the most useful metrics for evaluating homogenization techniques’ performance.

Secondarily, an evaluation of the influence of physical features (i.e., latitude, altitude, and distance from the sea) and artificial features (i.e., number of breaks, number of missing data) of the station data was performed through Pearson correlation. Regardless of the technique used, the quality of homogenization anti-correlates meaningfully to the number of breaks. Missing data do not seem to have any impact on HOMER homogenization in southern Sweden for both variables, and for maximum temperature in Slovenia, while a very weak, albeit significant, negative impact emerges between standard setup HOMER performance and number of missing data for minimum temperature in Slovenia. The reverse is true about ACMANT: the number of missing data significantly affects homogenization performance in a negative way, with the exception of minimum temperature homogenization for the Slovenia dataset.

Very weak, significant negative correlations are detected between station distance from the sea and factor of exceedance (*F*) and between station altitude and both *RMSE* and *F* for minimum temperature homogenization results obtained with the two HOMER techniques. This suggests that temperature at stations further from the sea and at higher altitude might be very slightly underestimated when homogenized with HOMER rather than with ACMANT. Latitude of the stations do not seem to have an impact on how well a technique homogenizes temperature data.

In general, the nature of the datasets (i.e., number of breaks and missing data) seems to have a more important role in yielding good homogenization results than physical parameters associated to the stations (i.e., latitude, elevation and distance from the sea).

Even though from this point of view, the skill of HOMER to replace most missing data give it the upper hand over ACMANT, the actual metrics show that ACMANT still performs better for these variables in these regions for what concerns *RMSE* and absolute error *B*^*abs*^, while HOMER performs better with regard to the factor of exceedance *F*.
